# Novel antidiabetic agents and the risk of respiratory diseases: a systematic review and meta-analysis of 27 randomized controlled trials

**DOI:** 10.3389/fmed.2025.1721311

**Published:** 2026-01-06

**Authors:** Zhexuan Yu, Bingyan Gu, Junyao Zhang, Weifeng Jin, Haitong Wan, Wei Jin

**Affiliations:** 1 School of Basic Medical Sciences, Zhejiang Chinese Medical University, Hangzhou, China; 2 The First School of Clinical Medicine, Zhejiang Chinese Medical University, Hangzhou, China; 3 The Second School of Clinical Medicine, Zhejiang Chinese Medical University, Hangzhou, China; 4 School of Pharmaceutical Sciences, Zhejiang Chinese Medical University, Hangzhou, China; 5 School of Medical Technology and Information Engineering, Zhejiang Chinese Medical University, Hangzhou, China

**Keywords:** SGLT2 inhibitors, GLP-1 receptor agonists, DPP-4 inhibitors, respiratory diseases, COPD, pneumonia

## Abstract

**Introduction:**

Patients with type 2 diabetes (T2D) are at increased risk of respiratory diseases, but the effects of novel glucose-lowering drugs on respiratory diseases remain unclear.

**Method:**

We conducted a systematic review and meta-analysis of 27 large randomized controlled trials (202,727 participants) assessing sodium-glucose cotransporter-2 inhibitors (SGLT2is), glucagon-like peptide-1 receptor agonists (GLP-1RAs), and dipeptidyl peptidase-4 inhibitors (DPP-4is). Prespecified outcomes included pneumonia, bronchitis, chronic obstructive pulmonary disease (COPD), pulmonary edema, pulmonary embolism, respiratory failure, and asthma. Pairwise and network meta-analyses were performed to estimate odds ratios (ORs) with 95% confidence intervals (CIs). This review was prospectively registered in PROSPERO (CRD420251158592).

**Result:**

Compared with placebo, SGLT2is significantly reduced the risk of six respiratory diseases: pneumonia (OR 0.84, 95% CI 0.77–0.92), bronchitis (OR 0.59, 95% CI 0.44–0.79), COPD (OR 0.76, 95% CI 0.64–0.90), pulmonary edema (OR 0.51, 95% CI 0.39–0.67), respiratory failure (OR 0.77, 95% CI 0.64–0.91), and asthma (OR 0.55, 95% CI 0.38–0.85). Benefits were broadly consistent in patients with and without T2D. GLP-1RAs were neutral in T2D but reduced pneumonia, respiratory failure, and asthma risk in obese populations. DPP-4is were largely neutral but increased asthma risk (OR 1.70, 95% CI 1.03–2.82).

**Conclusion:**

SGLT2 inhibitors showed robust respiratory protection that appeared largely independent of diabetes status, GLP-1 receptor agonists may provide benefit in obesity, and DPP-4 inhibitors offered limited advantage with a potential asthma risk. Our findings should be viewed as hypothesis generating, with concepts requiring validation in future studies.

**Systematic review registration:**

https://www.crd.york.ac.uk/prospero/display_record.php?ID=CRD420251158592, identifier PROSPERO (CRD420251158592).

## 1 Introduction

The global prevalence of type 2 diabetes (T2D) continues to rise, posing a major public health challenge (1). Beyond cardiovascular and renal complications, T2D has also been linked to an increased risk of respiratory diseases, further elevating hospitalization, mortality, and healthcare costs (2). Analyses from the Global Burden of Disease database indicate that seven respiratory conditions contribute substantially to global mortality (3), including infectious diseases (pneumonia, bronchitis) and non-infectious diseases (chronic obstructive pulmonary disease [COPD], pulmonary edema, pulmonary embolism, respiratory failure, and asthma).

In recent years, three major classes of novel glucose-lowering agents, including sodium-glucose cotransporter-2 inhibitors (SGLT2is), glucagon-like peptide-1 receptor agonists (GLP-1RAs), and dipeptidyl peptidase-4 inhibitors (DPP-4is), have attracted increasing attention for their pleiotropic effects beyond glucose control (4, 5). These drugs exert broad biological actions, including anti-inflammatory, antioxidative, and metabolic regulatory effects (6–9), which may modulate key pathological processes implicated in respiratory diseases (10, 11).

Although suggestive findings exist, clinical evidence on the respiratory effects of these agents remains limited and fragmented, largely derived from observational studies or subgroup analyses of individual trials with inconsistent results. For example, empagliflozin reduced pneumonia incidence in the EMPA-REG OUTCOME trial (12), whereas a dapagliflozin study did not show similar effects, although a potential benefit cannot be completely ruled out (13). Overall, large randomized controlled trials have primarily focused on cardiovascular, renal, or heart failure outcomes, with respiratory events reported only as secondary endpoints, lacking systematic synthesis.

To address this gap, we conducted a systematic review and meta-analysis of 27 large-scale randomized controlled trials (RCTs) to evaluate the effects of three major classes of glucose-lowering agents on the risks of seven respiratory diseases, with the aim of providing robust evidence to inform respiratory disease prevention and management in patients with diabetes.

## 2 Materials and methods

### 2.1 Search strategy and research selection

This systematic review and meta-analysis was conducted in accordance with the PRISMA guidelines and has been prospectively registered in PROSPERO (CRD420251158592) (14). A comprehensive search of PubMed and Embase was performed for eligible studies published up to January 8, 2025. The complete search strategies are provided in Supplementary Table 1. ClinicalTrials.gov was used in a complementary manner to verify trial registration numbers, retrieve detailed information on trial design and follow-up, and extract respiratory outcomes reported as endpoints for the randomized controlled trials identified through PubMed and Embase.

Trials were included if they met the following criteria:

1. Interventions: novel glucose-lowering drugs, including GLP-1RAs, DPP-4 inhibitors, and SGLT2 inhibitors;
2. Population: adults aged ≥19 years, including individuals with or without T2D who were enrolled in clinical trials reporting respiratory outcomes of interest;
3. Outcomes: reporting at least one respiratory outcome (pneumonia, bronchitis, COPD, pulmonary edema, pulmonary embolism, respiratory failure, asthma);
4. Study size: randomized controlled trials with ≥1,000 participants. Smaller trials (<1,000 participants) were excluded to reduce bias and increase statistical power.

### 2.2 Data extraction and quality assessment

Literature screening and study selection were performed by two independent reviewers according to predefined exclusion criteria. Duplicate publications were first eliminated, followed by an initial review of abstracts and titles to exclude irrelevant studies and those with small sample sizes. Subsequently, the remaining articles underwent eligibility assessment, with studies involving other interventions and incomplete results being excluded. Basic information, such as authors’ names, publication dates, sample sizes, patient ages, follow-up durations, and categories of medications in the intervention and control groups, was then extracted from the articles. The bias risk tool from Cochrane was utilized for the quality assessment of large randomized controlled trials, which included evaluations of selective outcome reporting, sequence generation, blinding, incomplete outcome data, bias risk categorization (unclear, high, or low), and allocation concealment.

Study selection, data extraction, and quality assessment were conducted by 2 independent authors. Any disagreement was resolved by discussion until consensus was reached, or by consulting a third author. To reduce heterogeneity and improve interpretability, we restricted the analyses to seven prespecified respiratory outcomes (pneumonia, bronchitis, COPD, pulmonary edema, pulmonary embolism, respiratory failure, and asthma). Other respiratory events not belonging to these categories or with fewer than 200 total cases (e.g., interstitial lung disease, pulmonary fibrosis, nonspecific respiratory symptoms) were excluded from pooled analyses.

### 2.3 Statistical analysis

The ultimate results of the meta-analysis are presented using 95% confidence intervals and odds ratios as effect measures. The assessment of heterogeneity is conducted using the *I*^2^ statistic and *p*-value. When significant heterogeneity is detected, a random-effects model is applied, whereas a fixed-effects model is employed in the absence of heterogeneity. Heterogeneity levels are categorized by the *I*^2^ statistic as follows: 0–25% indicates no heterogeneity, 25–50% indicates low heterogeneity, 50–75% indicates moderate heterogeneity, and 75–100% indicates high heterogeneity. A *p*-value below 0.1 is also considered indicative of the presence of heterogeneity. Funnel plots are used to publish and assess bias (15). Egger’s linear regression test and Begg’s rank correlation test are additionally applied to quantitatively evaluate small-study effects and funnel plot asymmetry.

Since none of the network diagrams are closed, the inconsistency of the network (i.e., statistical differences between direct and indirect results) cannot be evaluated. In the test for the overall effect, a *P_Effect_* value greater than 0.05 is considered to indicate that the result is not statistically significant.

To further evaluate the comparative effects of these three novel antidiabetic agents on disease risk, a frequentist network meta-analysis was performed. The network meta-analysis was conducted to assess the comparative impacts of GLP-1RAs, DPP-4 inhibitors, and SGLT2 inhibitors on the risk of respiratory diseases, and the safest interventions were ranked according to the SUCRA (16). The pairwise meta-analysis was employed to uncover the relationship between new hypoglycemic drugs and the risk of respiratory diseases. Through experimental screening, 21 trials involving patients with T2D and 6 trials involving patients both with and without T2D were included. The meta-analysis was performed using STATA/MP 17.0, while bias risk assessment was carried out using Review Manager 5.3.

## 3 Results

### 3.1 Study selection, baseline characteristics, and risk of bias

**Figure 1:**
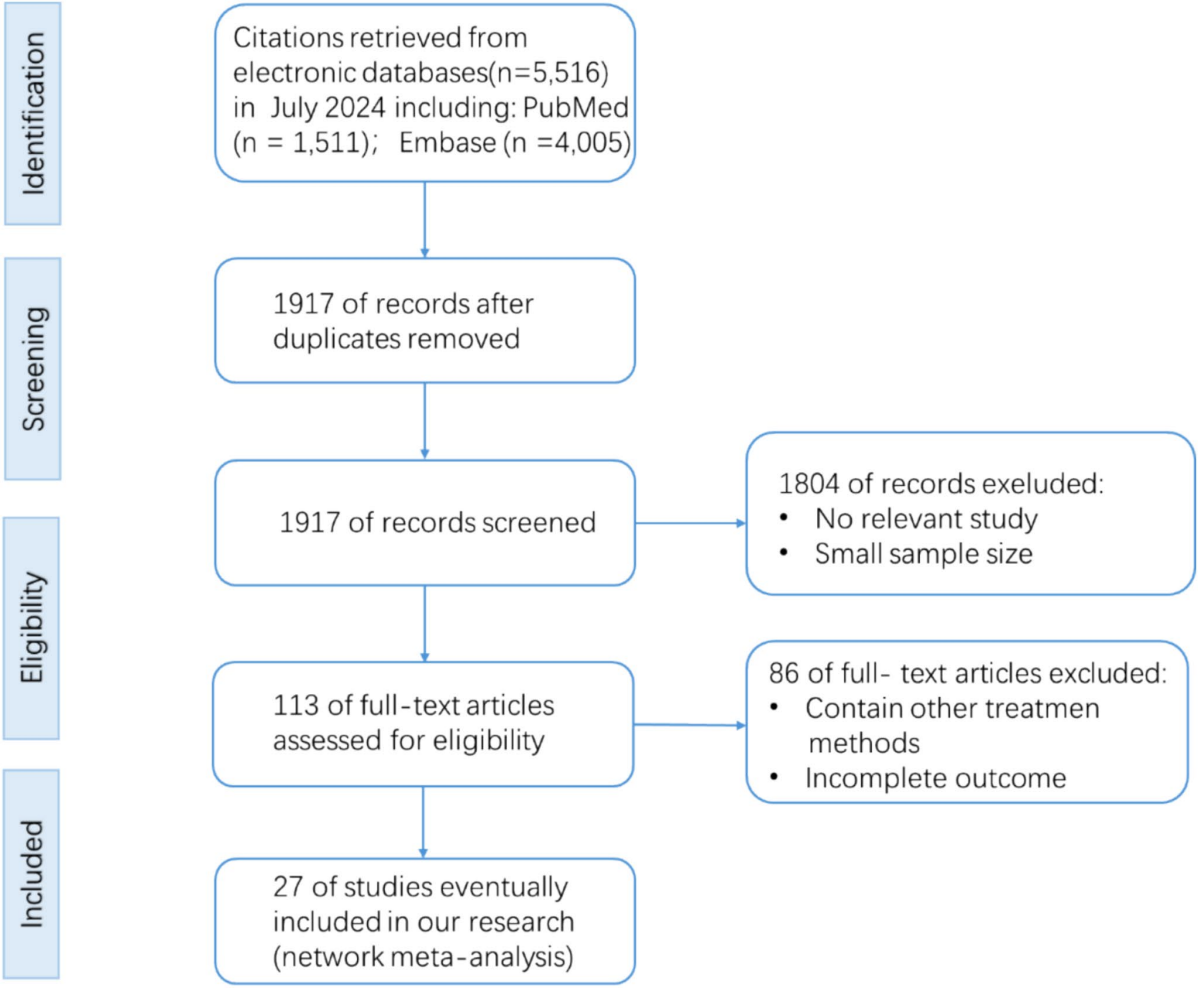
Flowchart of literature screening.

A total of 27 large-scale RCTs were included, comprising 202,727 participants; the study selection process is shown in Figure 1. The included RCTs were: NCT00968708 (17), NCT01107886 (18), NCT00790205 (19), NCT01703208 (20), NCT01897532 (21), NCT01243424 (22), NCT01147250 (23), NCT01179048 (24), NCT01720446 (25), NCT01144338 (26), NCT02465515 (27), NCT01394952 (28), NCT02692716 (29), NCT03496298 (30), NCT01131676 (31), NCT01730534 (32), NCT01989754 (33), NCT01032629 (34), NCT02065791 (35), NCT01986881 (36), NCT03315143 (37), NCT03036124 (38), NCT03036150 (39), NCT03057977 (40), NCT03057951 (41), NCT03619213 (42), NCT03594110 (43).

With respect to study populations, trials of DPP-4 inhibitors and GLP-1 receptor agonists enrolled only patients with T2D, whereas trials of SGLT2 inhibitors comprised two types of cohorts: (1) patients with T2D (7 trials) and (2) individuals with or without T2D (6 trials). Trial registration numbers and baseline characteristics are provided in Table 1, and HbA1c, BMI, and eGFR data are summarized in Supplementary Table 2. The mean age of participants was 64.8 years (range 60.3–71.8 years). All studies were placebo-controlled, except for CAROLINA, which used glimepiride as an active comparator.

**Table 1:** Baseline characteristics table.

First author (year)	Study ID ClinicalTrials.gov	Name	Patients	Intervention	Control	Follow -up (years)	Number of patients	Age (years)
White (2013)	NCT00968708 (17)	EXAMI NE	T2D and ACS	Alogliptin	Placebo	1.5	5,380	60.9
Scirica (2013)	NCT01107886 (18)	SAVOR-TIMI 53	T2D and CVD	Saxagliptin	Placebo	2.1	16,492	65.1
Green (2015)	NCT00790205 (19)	TECOS	T2D and CVD	Sitagliptin	Placebo	3.0	14,671	65.5
Gantz (2017)	NCT01703208 (20)	OMNEON	T2D and CVD	Omarigliptin	Placebo	1.8	4,192	64.0
Rosenstock (2019)a	NCT01897532 (21)	CARMELINA	T2D and CVD	Linagliptin	Placebo	2.2	6,979	66.0
Rosenstock (2019)b	NCT01243424 (22)	CAROLINA	T2D and CAD	Linagliptin	Glimeride	6.3	6,033	64
Pfeffer (2015)	NCT01147250 (23)	ELIXA	T2D and CAD	Lixisenatide	Placebo	2.1	6,063	60.3
Marso (2016)a	NCT01179048 (24)	LEADE R	T2D and CVD	Liraglutide	Placebo	3.8	9,340	64.3
Marso (2016)b	NCT01720446 (25)	SUSTAI N-6	T2D and CVD and CKD	Semaglutide	Placebo	2.1	3,297	64.6
Holman (2017)	NCT01144338 (26)	EXSCEL	T2D and CVD	Exenatide	Placebo	3.2	14,752	62.0
Hernandez (2018)	NCT02465515 (27)	HARMONY	T2D and CVD	Albiglutide	Placebo	1.6	9,432	64.0
Gerstein (2019)	NCT01394952 (28)	REWIND	T2D and CVD	Dulaglutide	Placebo	5.4	9,901	66.0
Husain (2019)	NCT02692716 (29)	PIONEER-6	T2D and CVD	Semaglutide	Placebo	1.3	3,183	66.0
Gerstern (2021)	NCT03496298 (30)	AMPLITUDE-O	T2D and CVD	Efpeglenatide	Placebo	1.8	4,076	64.5
Wanner (2016)	NCT01131676 (31)	EMPA- REG OUTC OME	T2D and CVD	Empagliflozin	Placebo	3.1	7,020	63.1
Wiviott (2018)	NCT01730534 (32)	DECLARE–TIMI58	T2D and ASCVD	Dapagliflozin	Placebo	4.2	17,161	63.9
Mahaffey (2018)a	NCT01989754 (33)	CANVAS-R	T2D and CVD	Canagliflozin	Placebo	3.6	5,807	63.5
Mahaffey (2018)b	NCT01032629 (34)	CANVAS	T2D and CVD	Canagliflozin	Placebo	3.6	4,330	63.5
Perkovic (2019)	NCT02065791 (35)	CREDENCE	T2D and CK	Canagliflozin	Placebo	2.6	4,401	62.9
Cannon (2020)	NCT01986881 (36)	VERTIS-CV	T2D and CVD	Ertugliflozin	Placebo	3.5	8,246	64.4
Bhatt (2021)a	NCT03315143 (37)	SCORED	T2D and CKD	Sotagliflozin	Placebo	1.3	10,584	69.0
McMurray (2019)	NCT03036124 (38)	DAPA-HF	HFmrEF	Dapagliflozin	Placebo	1.5	4,744	66.2
Heerspink (2020)	NCT03036150 (39)	DAPA-CKD	CVD and CKD	Dapagliflozin	Placebo	2.3	4,304	62.0
Packer (2020)	NCT03057977 (40)	EMPEROR-Reduced 2020	CVD and HF	Empagliflozin	Placebo	1.3	3,730	67.2
Anker (2021)	NCT03057951 (41)	EMPEROR-Preserved	HFmrEF	Empagliflozin	Placebo	2.1	5,988	71.8
Peikert (2022)	NCT03619213 (42)	DELIVER	HFmrEF	Dapagliflozin	Placebo	2.3	6,263	71.7
Herrington (2023)	NCT03594110 (43)	EMPA-KIDNEY	CVD and CKD	Empagliflozin	Placebo	2.0	6,609	63.9

Seven respiratory outcomes were prespecified: infectious diseases (pneumonia, bronchitis) and non-infectious diseases (COPD, pulmonary edema [acute or chronic], pulmonary embolism, respiratory failure [acute or chronic], and asthma). These events were primarily reported as secondary outcomes on ClinicalTrials.gov, rather than being predefined primary outcomes of the original trials. Outcomes with fewer than 200 events were not included in pooled analyses. Some trials did not report all outcomes: for example, CANVAS did not report pulmonary embolism, respiratory failure, bronchitis, or asthma, and EXSCEL did not report pulmonary embolism; all other trials reported the full set of respiratory outcomes.

Risk of bias was assessed using the Cochrane tool, with overall judgments summarized in Supplementary Figure 2. The interventions and trial numbers contributing to the network meta-analysis are presented in Supplementary Figure 1. Overall study quality was acceptable, although selective reporting and unclear risk were noted in a minority of domains.

### 3.2 Primary outcomes by drug class

**Figure 2:**
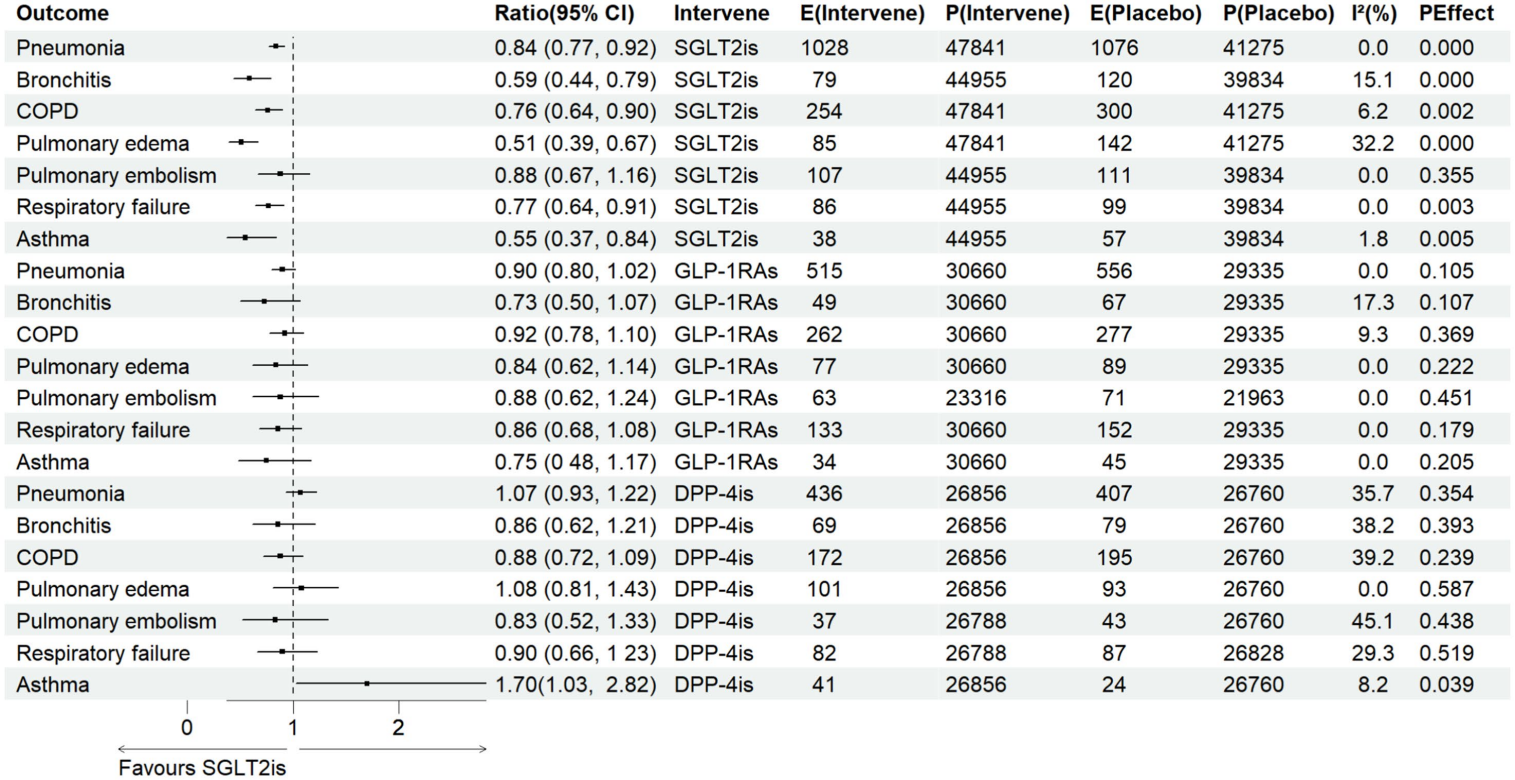
Results of a meta-analysis of six respiratory diseases.

The paired meta-analysis results for the seven respiratory diseases are illustrated in Figure 2. In direct comparisons with placebo, SGLT2 inhibitors significantly reduced the risk of multiple respiratory diseases. For infectious diseases, the pooled effects were: pneumonia OR 0.84 (95% CI 0.77–0.92; *p* < 0.001; *I*^2^ = 0.0%) and bronchitis OR 0.59 (95% CI 0.44–0.79; *p* < 0.001; *I*^2^ = 15.1%). For non-infectious diseases, significant effects were observed for COPD OR 0.76 (95% CI 0.64–0.90; *p* = 0.001; *I*^2^ = 6.2%), pulmonary edema OR 0.51 (95% CI 0.39–0.67; *p* < 0.001; *I*^2^ = 33.3%), respiratory failure OR 0.77 (95% CI 0.64–0.91; *p* = 0.002; *I*^2^ = 0.0%), and asthma OR 0.55 (95% CI 0.38–0.85; *p* = 0.005; *I*^2^ = 1.8%). All overall effect tests reached statistical significance.

In comparison to placebo, GLP-1 receptor agonists showed trends toward reduced risks for two infectious and five non-infectious respiratory diseases, but none reached statistical significance. Although no heterogeneity was detected, the wide confidence intervals limited the certainty of these findings.

Compared with placebo, DPP-4 inhibitors showed generally neutral associations across respiratory outcomes, with non-significant trends toward reduced risks of pneumonia, pulmonary embolism, respiratory failure, and bronchitis. In contrast, DPP-4 inhibitors were associated with a significantly increased risk of asthma (OR 1.70, 95% CI 1.03–2.82; *p* = 0.039; *I*^2^ = 8.2%), without evidence of heterogeneity.

The network meta-analysis results, including forest plots, bias assessment, and SUCRA rankings for the seven respiratory outcomes, are presented in Supplementary Figures 3–9. SGLT2 inhibitors were associated with lower odds of pneumonia compared with DPP-4 inhibitors and placebo. Both SGLT2 inhibitors and GLP-1 receptor agonists showed lower odds of asthma compared with DPP-4 inhibitors and placebo, while SGLT2 inhibitors also demonstrated lower odds of COPD, respiratory failure, and bronchitis versus placebo. SUCRA rankings further indicated that SGLT2 inhibitors were most likely to be the most effective intervention for six respiratory outcomes. There was no evidence of small-study or publication bias, as neither Egger’s test nor Begg’s test indicated significant small-study effects for outcomes with sufficient trials (all *p* ≥ 0.05), consistent with the largely symmetric funnel plots.

### 3.3 Subgroup and exploratory analyses

#### 3.3.1 Analyses by diabetes status

The experimental cohort receiving SGLT2 inhibitors included two groups: (1) patients with T2D and (2) patients with or without T2D. Thirteen trials were analyzed, comprising 7 restricted to T2D patients and 6 enrolling individuals with or without T2D. In the latter, approximately half of the participants had T2D and half did not (48.8% vs. 51.2%, based on aggregated totals; Supplementary Table 5). The effects of SGLT2 inhibitors on seven respiratory diseases in these groups are shown in Supplementary Figures 3–9 (subgroup analysis), and summarized in Table 2.

**Table 2:** Effects of SGLT2 inhibitors on the risk of 7 respiratory diseases in two groups of patients.

Respiratory diseases	With T2D	With or without T2D	*p* for interaction
COPD	0.77 (0.62, 0.95)	0.72 (0.55, 0.94)	0.701
Pneumonia	0.83 (0.73, 0.93)	0.86 (0.76, 0.98)	0.609
Pulmonary edema	0.42 (0.29, 0.61)	0.78 (0.51, 1.19)	0.036
Pulmonary embolism	0.89 (0.63, 1.24)	0.86 (0.53, 1.38)	0.912
Respiratory failure	0.79 (0.64, 0.98)	0.71 (0.53, 0.96)	0.567
Bronchitis	0.56 (0.39, 0.79)	0.59 (0.35, 0.97)	0.876
Asthma	0.52 (0.32, 0.85)	0.65 (0.30, 1.38)	0.631

The values in the table represent the 95% confidence interval (CI) and odds ratio (OR). An OR is considered not statistically significant when its 95% CI includes 1.

SGLT2 inhibitors significantly reduced the risk of COPD, pneumonia, respiratory failure, and bronchitis in both groups, with similar odds ratios and confidence intervals, suggesting consistent protective effects irrespective of T2D status. For pulmonary embolism, the confidence intervals crossed 1 in both groups, indicating no significant effect. Protective associations with pulmonary edema and asthma were observed only in patients with T2D, whereas no significant effects were detected in mixed populations; notably, the subgroup interaction was significant for pulmonary edema (*p* = 0.036) but not for asthma (*p* = 0.631).

Collectively, these findings demonstrate that SGLT2 inhibitors confer robust protection against several major respiratory diseases regardless of T2D status, while benefits for pulmonary edema appear confined to patients with T2D.

#### 3.3.2 Drug-specific analyses

**Figure 3:**
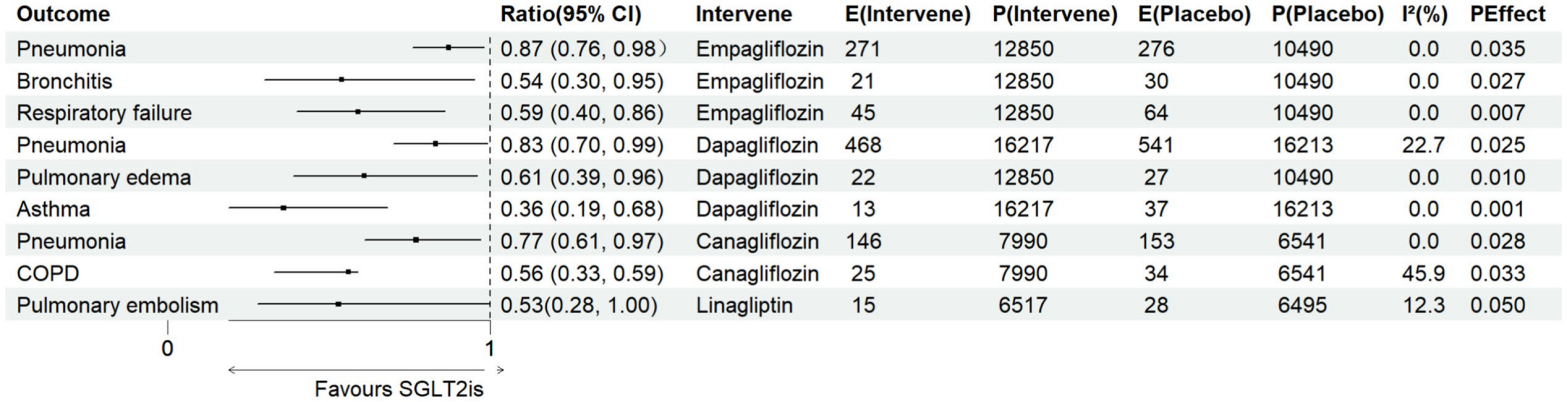
Summary of positive results from the subgroup meta-analysis.

To explore potential heterogeneity within drug classes, we performed drug-specific subgroup analyses restricted to agents with sufficient trial data to allow pooled estimates. The effects of linagliptin, semaglutide, empagliflozin, dapagliflozin, and canagliflozin on seven respiratory outcomes were assessed. Detailed results of pairwise meta-analyses are presented in Supplementary Figures 3–9, with a summary of significant findings shown in Figure 3.

1. Linagliptin: A borderline protective effect was observed for pulmonary embolism (OR = 0.53, 95% CI 0.28–1.00; PEffect = 0.050; *I*^2^ = 12.3%), while no significant associations were found for other respiratory diseases.
2. Semaglutide: No significant impact was detected across the seven outcomes.
3. Empagliflozin: Consistent protective effects were observed across both infectious and non-infectious diseases. Significant reductions were noted for pneumonia (OR = 0.87, 95% CI 0.76–0.98; *P_Effect_* = 0.034; *I*^2^ = 0.0%), bronchitis (OR = 0.54, 95% CI 0.30–0.95; *P_Effect_* = 0.027; *I*^2^ = 0.0%), and respiratory failure (OR = 0.59, 95% CI 0.40–0.86; *P_Effect_* = 0.007; *I*^2^ = 0.0%).
4. Dapagliflozin: Protective effects were observed against both infectious and non-infectious conditions, including pneumonia (OR = 0.83, 95% CI 0.70–0.99; *P_Effect_* = 0.025; *I*^2^ = 22.7%), pulmonary edema (OR = 0.61, 95% CI 0.39–0.96; *P_Effect_* = 0.010; *I*^2^ = 0.0%), and asthma (OR = 0.36, 95% CI 0.19–0.68; *P_Effect_* = 0.001; *I*^2^ = 0.0%).
5. Canagliflozin: Significant risk reduction was observed for pneumonia (OR = 0.77, 95% CI 0.61–0.97; *P_Effect_* = 0.028; *I*^2^ = 0.0%). For COPD, a favorable but heterogeneous association was noted (OR = 0.56, 95% CI 0.33–0.59; *P_Effect_* = 0.033; *I*^2^ = 45.9%).

To further evaluate comparative efficacy, a network meta-analysis was performed including semaglutide, dapagliflozin, empagliflozin, and canagliflozin (linagliptin was excluded because the Rosenstock 2019 trial used glimepiride as the comparator). Forest plots indicated that dapagliflozin, empagliflozin, and canagliflozin significantly reduced the risk of pneumonia relative to placebo, with dapagliflozin additionally lowering asthma risk and empagliflozin reducing respiratory failure risk. Funnel plots suggested no major publication bias.

Surface under the cumulative ranking curves (SUCRA) were calculated to estimate the probability of each intervention being the safest option (Table 3). Results indicated that canagliflozin ranked highest for COPD and pneumonia, empagliflozin for respiratory failure, and dapagliflozin for asthma. These rankings were consistent with the pairwise meta-analysis findings, reinforcing the robustness of drug-specific differences. While the SUCRA rankings suggest relative differences in the probability of benefit among the included interventions, these values should be interpreted within the context of the network meta-analysis framework and do not indicate direct clinical superiority of one drug over another.

**Table 3:** SUCRA results for each intervention drug in the subgroup analysis.

Respiratory diseases	Semaglutide	Dapagliflozin	Empagliflozin	Canagliflozin	PBO
COPD	20.6	56.0	68.9	83.6	20.8
Pneumonia	65.2	46.4	58.4	76.5	3.6
Pulmonary edema	25.7	69.2	56.5	74.4	24.3
Pulmonary embolism	57.1	56.4	79.7	13.2	43.6
Respiratory failure	54.4	47.8	84.8	49.9	13.1
Bronchitis	66.5	43.9	65.6	61.4	12.6
Asthma	81.8	82.5	42.7	10.7	32.3

The color from dark to light represents the SUCRA result from large to small, and the color order from large to small is blue, orange, yellow, dark green, light green.

#### 3.3.3 Exploratory analyses of GLP-1 receptor agonists in obesity trials

In analyses restricted to T2D populations, GLP-1 receptor agonists (GLP-1RAs) showed consistent trends toward reduced risks across all seven respiratory outcomes compared with placebo; however, none reached statistical significance (all *p* > 0.05), and confidence intervals were wide.

To expand the sample size and assess effects in obesity populations, three large-scale obesity trials were incorporated while keeping other eligibility criteria unchanged: STEP 2 (44), Wilding (45), and SELECT (46). STEP 2 enrolled patients with T2D and obesity, whereas Wilding and SELECT included individuals with obesity alone. Baseline characteristics are shown in Supplementary Table 4.

Pooled analyses including these trials demonstrated significant risk reductions for one infectious disease and three non-infectious diseases, with no evidence of heterogeneity: pneumonia (OR = 0.86, 95% CI 0.77–0.96; *N* = 10 trials; *P_Effect_* = 0.050; *I*^2^ = 12.3%), respiratory failure (OR = 0.78, 95% CI 0.63–0.95; *N* = 9 trials; *P_Effect_* = 0.014; *I*^2^ = 0.0%), bronchitis (OR = 0.70, 95% CI 0.49–0.98; *N* = 9 trials; *P_Effect_* = 0.038; *I*^2^ = 0.0%), and asthma (OR = 0.63, 95% CI 0.42–0.93; *N* = 8 trials; *P_Effect_* = 0.022; *I*^2^ = 0.0%). Results are shown in Supplementary Figure 10.

**Figure 4:**
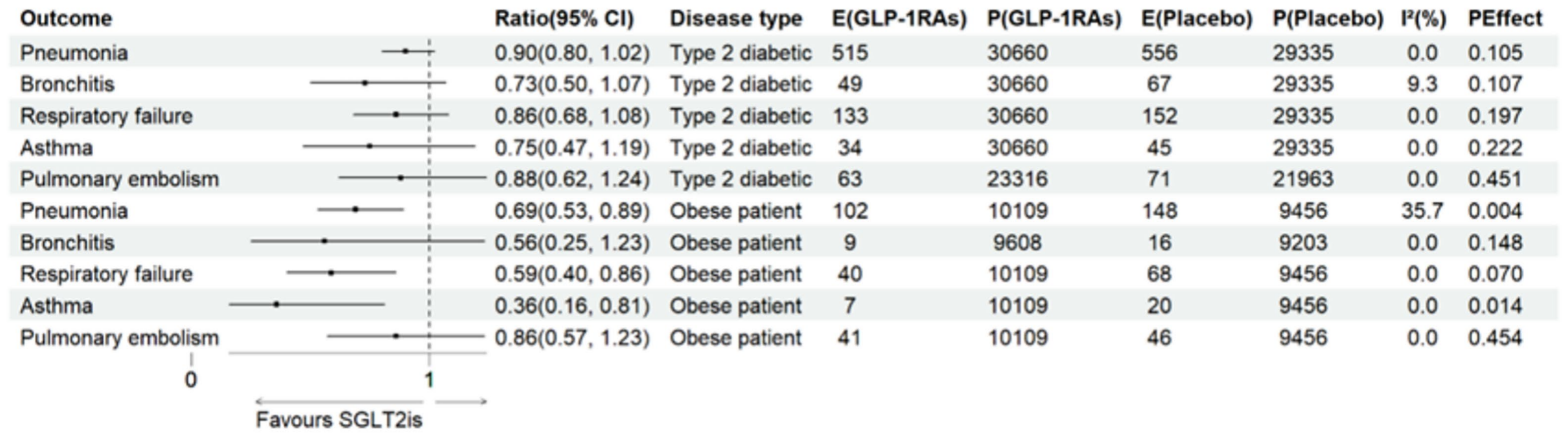
The impact of GLP-1RAs on respiratory diseases in two types of patients.

For population-specific analyses, STEP 2 was excluded to avoid confounding, as it enrolled both T2D and obese patients. Figure 4 provides a visual summary of these results. In obese individuals, GLP-1RAs significantly reduced the risk of pneumonia, respiratory failure, and asthma compared with placebo, with narrow confidence intervals and no heterogeneity. In T2D populations, no statistically significant associations were observed across five respiratory outcomes. These differential findings may be partly explained by the substantially higher baseline burden of systemic inflammation, central adiposity, and respiratory mechanical loading in obese individuals, which could amplify the impact of GLP-1RAs on weight reduction and airway inflammation. Detailed outcome-specific forest plots are presented in Supplementary Figure 11. Given that these analyses were exploratory, based on trials in which respiratory events were secondary outcomes, the findings should be interpreted as hypothesis-generating.

## 4 Discussion

This meta-analysis systematically evaluated the impact of three major classes of novel glucose-lowering drugs—SGLT2 inhibitors, GLP-1 receptor agonists, and DPP-4 inhibitors—on the risk of seven clinically relevant respiratory diseases. By integrating data from 27 large-scale randomized controlled trials encompassing more than 200,000 participants, we provide new insights into the potential respiratory benefits of these agents from a drug-repurposing perspective.

### 4.1 Principal findings

Our results demonstrate that SGLT2 inhibitors confer robust protective effects across both infectious (pneumonia, bronchitis) and non-infectious (COPD, respiratory failure) respiratory outcomes. These benefits were observed in different populations, consistent between patients with T2DM and individuals without T2DM. This suggests that the respiratory protection of SGLT2 inhibitors is largely independent of diabetic status, although the modifying effect of glycemic control cannot be completely ruled out. However, a T2D-specific benefit was observed only for pulmonary edema. One possible explanation is that patients with T2D have a higher baseline risk of fluid retention and volume overload, which may amplify the decongestive effects of SGLT2 inhibitors. At the drug level, empagliflozin, dapagliflozin, and canagliflozin showed complementary profiles of benefit, reinforcing the class effect of SGLT2 inhibitors.

By contrast, GLP-1 receptor agonists exhibited neutral overall effects in T2D populations, with wide confidence intervals and no significant associations for most outcomes. In analyses restricted to T2D populations, GLP-1 receptor agonists (GLP-1RAs) showed consistent trends toward reduced risks across all seven respiratory outcomes compared with placebo; however, none reached statistical significance (all *p* > 0.05), and wide confidence intervals limited the precision of estimates. Previous large-scale studies including 1,223,130 patients reported that SGLT-2 inhibitors and GLP-1RAs were associated with reduced COPD risk compared with sulfonylureas (47). By contrast, our trial population comprised only 202,727 participants, which may have reduced statistical power. Similar neutral findings of GLP-1RAs on respiratory outcomes have been reported in 2021 (48), while a 2022 analysis including both obese and T2D individuals suggested protective effects only for pulmonary edema and bronchitis, with wide confidence intervals (49). Given that GLP-1RAs also induce substantial weight loss and obesity is a major risk factor for respiratory diseases, further evaluation in obese populations was warranted.

The inclusion of large cohorts with obesity in additional analyses highlighted potential benefits in terms of reduced risk of pneumonia and asthma. Although these results are encouraging, they remain preliminary and are critically important to confirm in specifically designed, prospective clinical trials.

DPP-4 inhibitors did not demonstrate significant associations with respiratory outcomes overall, although individual agents such as linagliptin showed possible protective effects against pulmonary embolism in subgroup analyses. Notably, DPP-4 inhibitors were associated with an increased risk of asthma, which warrants clinical caution. Given the limited signal strength, these observations should be considered hypothesis-generating.

### 4.2 Potential mechanisms

Several biological mechanisms may explain the observed findings. SGLT2 inhibitors not only improve glycemic control but also increase hematocrit (50, 51), reduce systemic congestion, and attenuate inflammatory pathways such as NLRP3 inflammasome activation. These effects could plausibly account for the observed reductions in pneumonia, pulmonary edema, and respiratory failure (52, 53). GLP-1 receptor agonists are known to reduce airway inflammation, inhibit airway remodeling, and modulate mucus secretion (54). Obesity is strongly associated with low-grade systemic inflammation, increased central adiposity, and mechanical loading on the respiratory system, all of which contribute to a higher baseline risk of pneumonia, respiratory failure and asthma. Their stronger effects in obesity trials may relate to the dual impact on body weight and systemic inflammation, both key drivers of respiratory morbidity (55). DPP-4 inhibitors reduce oxidative stress and inflammatory cytokine release, but their modest efficacy may explain the lack of significant clinical associations in our analyses (56, 57).

### 4.3 Comparison with previous studies

Our findings extend prior observational and experimental reports linking SGLT2 inhibitors and GLP-1 receptor agonists with improved pulmonary outcomes. Unlike earlier studies, which were often underpowered or limited to single respiratory endpoints, we systematically synthesized evidence across seven outcomes using high-quality RCT data. Notably, our identification of obesity as a potential modifier of GLP-1RA efficacy adds a novel dimension to the current literature.

### 4.4 Clinical implications

These results suggest that SGLT2 inhibitors may offer clinically meaningful respiratory protection, independent of diabetes status, supporting their broader therapeutic value beyond cardiovascular and renal outcomes. From a clinical standpoint, these signals indicate that respiratory considerations may serve as an additional factor when individualizing therapy for patients at elevated risk of pulmonary complications. For GLP-1 receptor agonists, our exploratory findings highlight the need for dedicated respiratory outcome trials, particularly in obese populations where benefits appear more pronounced. These preliminary observations also underscore the potential for weight- and inflammation-mediated respiratory advantages to influence treatment decisions in selected high-risk groups. Although DPP-4 inhibitors did not show consistent protective effects, their favorable safety profile warrants further mechanistic studies. Collectively, these observations provide a rationale for considering respiratory benefits in the long-term risk–benefit assessment of novel antidiabetic agents. Looking ahead, future research should prioritize trials with prespecified respiratory endpoints and patient-level analyses to validate these signals and determine which subpopulations are most likely to benefit.

### 4.5 Limitations

There are several limitations in this meta-analysis. First, respiratory diseases were not prespecified as primary outcomes in the included trials; they were reported as secondary outcomes on ClinicalTrials.gov, introducing inevitable risks of reporting and ascertainment bias. Outcome definitions may also have varied across trials. Second, the absence of centrally adjudicated endpoints limits the consistency of event classification. Third, patient-level data were not available, restricting subgroup analyses to the trial level and precluding detailed adjustment for confounders. Fourth, relatively small event counts for several outcomes reduced statistical power, meaning that estimates and heterogeneity tests (*I*^2^ and *p*-values) should be interpreted with caution. Fifth, subgroup and exploratory analyses, particularly those involving obesity trials, remain hypothesis-generating given limited sample sizes. In addition, the SUCRA-based rankings should be considered hypothesis-generating, given the absence of head-to-head comparisons and variability across the included trials. Finally, heterogeneity across trial populations, drug dosing, and follow-up durations could not be fully addressed.

## 5 Conclusion

In summary, this comprehensive meta-analysis demonstrated that SGLT2 inhibitors consistently reduced the risk of multiple respiratory diseases, with effects broadly independent of diabetes status. GLP-1 receptor agonists were neutral in T2D but showed potential benefits in obese individuals, suggesting that metabolic phenotype may influence treatment responsiveness. DPP-4 inhibitors did not show consistent protective effects. These hypothesis-generating findings merit confirmation in future large-scale studies with prespecified, adjudicated respiratory outcomes and patient-level data to establish causal relationships and guide clinical translation.

## Data Availability

The datasets presented in this study can be found in online repositories. The names of the repository/repositories and accession number(s) can be found in the article/Supplementary material.
