# Supplementary material for: Novel antidiabetic agents and the risk of respiratory diseases: a systematic review and meta-analysis of 27 randomized controlled trials
Source: Front Med (Lausanne). 2026 Jan 6;12:1721311. doi: 10.3389/fmed.2025.1721311 (PMC12815760; doi:10.3389/fmed.2025.1721311)
Supplement: Supplementary file 1 [file Supplementary_file_1.docx]

Supplementary materials

**Supplementary Table 1: Search policy**

| Pubmed Search terms |
| --- |
| ((‘Sodium-Glucose Transporter 2 Inhibitors’[MeSH] OR ‘Sodium Glucose cotransporter’ OR ‘Sodium-dependent glucose cotransporter’ OR ‘Sodium Glucose transporter’ OR SGLT2 OR SGLT-2 OR ‘SGLT-2‘ OR empagliflozin OR dapagliflozin OR canagliflozin OR sotagliflozin OR luseogliflozin OR ipragliflozin OR remogliflozin OR sergliflozin OR ertugliflozin OR tofogliflozin OR ‘Dipeptidyl-Peptidase IV Inhibitors’ [MeSH] OR ‘Dipeptidyl-Peptidase IV Inhibitor’ OR ‘Dipeptidyl peptidase 4 inhibitor’ OR ‘DPP-4 inhibitor*’ OR Gliptins OR DPP-4i OR sitagliptin OR vildagliptin OR saxagliptin OR linagliptin OR gemigliptin OR teneligliptin OR alogliptin OR trelagliptin OR evogliptin OR gosogliptin OR dutogliptin OR omarigliptin OR ‘Glucagon-Like Peptide-1 Receptor’[Mesh] OR ‘Glucagon-like peptide 1 receptor agonist*’ OR ‘GLP-1 receptor agonist*’ OR ‘GLP-1 agonist*‘ OR ‘GLP-1RA’ OR exenatide OR liraglutide OR lixisenatide OR albiglutide OR dulaglutide OR semaglutide) AND (‘Diabetes Mellitus, Type 2’[Mesh] OR ‘Type 2 diabetes’ OR T2DM OR T2D) AND ("Randomized Controlled Trial"[Publication Type] OR random OR RCT OR placebo OR trial) AND (Cardiovascular OR renal OR CVOT OR 'CVOT' OR HF OR CKD OR ‘Pulmonary Disease, Chronic Obstructive’[MeSH] OR ‘Chronic Obstructive Airway Disease’ OR ‘Chronic Obstructive Lung Disease’ OR ‘COAD’ OR ‘Chronic Obstructive Pulmonary Diseases’ OR ‘COPD’ OR ‘Chronic Obstructive Pulmonary Disease’ OR ‘Airflow Obstructions, Chronic’ OR ‘Airflow Obstruction, Chronic’ OR ‘Chronic Airflow Obstruction’ OR ‘Chronic Airflow Obstructions’ OR ‘Chronic Obstructive Pulmonary Disease’ OR ‘Pneumonias’[MeSH] OR ‘Lobar Pneumonia’ OR ‘Lobar Pneumonias’ OR ‘Pneumonias, Lobar’ OR ‘Pneumonia, Lobar’ OR ‘Experimental Lung Inflammation’ OR ‘Inflammation, Experimental Lung’ OR ‘Lung Inflammation’ OR ‘Pulmonary Inflammations’ OR Inflammation, Lung OR ‘pulmonary embolism’[MeSH] OR ‘Embolism, Pulmonary’ OR ‘Pulmonary Thromboembolisms’ OR ‘Thromboembolism, Pulmonary’ OR ‘Thromboembolisms, Pulmonary’ OR ‘Pulmonary Edema’[MeSH] OR ‘Wet Lung OR Lung, Wet’ OR ‘Pulmonary Edemas’ OR ‘Edema, Pulmonary’ OR 'Respiratory Insufficiency'[MeSH] OR Acute Hypercapnic Respiratory Failure OR Hypercapnic Acute Respiratory Failure OR Respiratory Failure OR Hypoxemic Respiratory Failure OR Failure, Hypercapnic Respiratory OR Respiratory Depression OR 'Bronchitis'[MeSH] OR Bronchiolitis, Viral OR Bronchiolitis OR 'Asthma'[MeSH] OR Asthmas OR Bronchial Asthma OR Asthma, Bronchial) AND (alladult[Filter]) AND (english[Filter])) 1571 |
| Embase |
| (('Sodium-Glucose Transporter 2 Inhibitors*' or 'Sodium Glucose cotransporter' or 'Sodium-dependent glucose cotransporter' or 'Sodium Glucose transporter' or SGLT2 or SGLT-2 or 'SGLT-2' or empagliflozin or dapagliflozin or canagliflozin or sotagliflozin or luseogliflozin or ipragliflozin or remogliflozin or sergliflozin or ertugliflozin or tofogliflozin or 'Dipeptidyl-Peptidase IV Inhibitors*' or 'Dipeptidyl-Peptidase IV Inhibitor' or 'Dipeptidyl peptidase 4 inhibitor*' or 'DPP-4 inhibitor*' or Gliptins or DPP-4i or sitagliptin or vildagliptin or saxagliptin or linagliptin or gemigliptin or teneligliptin or alogliptin or trelagliptin or evogliptin or gosogliptin or dutogliptin or omarigliptin or 'Glucagon-Like Peptide-1 Receptor*' or 'Glucagon-like peptide 1 receptor agonist*' or 'GLP-1 receptor agonist' or 'GLP-1 agonist*' or 'GLP-1RA' or exenatide or liraglutide or lixisenatide or albiglutide or dulaglutide or semaglutide) and ('Diabetes Mellitus, Type 2*' or 'Type 2 diabetes' or T2DM or T2D) and ("Randomized Controlled Trial" or random or RCT or placebo or trial) and (Cardiovascular or renal or CVOT or 'CVOT' or HF or CKD or 'Pulmonary Disease, Chronic Obstructive*' or 'Chronic Obstructive Airway Disease' or 'Chronic Obstructive Lung Disease' or 'COAD' or 'Chronic Obstructive Pulmonary Diseases' or 'COPD' or 'Chronic Obstructive Pulmonary Disease' or 'Airflow Obstructions, Chronic' or 'Airflow Obstruction, Chronic' or 'Chronic Airflow Obstruction' or 'Chronic Airflow Obstructions' or 'Chronic Obstructive Pulmonary Disease' or 'Pneumonias*' or 'Lobar Pneumonia' or 'Lobar Pneumonias' or 'Pneumonias, Lobar' or 'Pneumonia, Lobar' or 'Experimental Lung Inflammation' or 'Inflammation, Experimental Lung' or 'Lung Inflammation' or 'Pulmonary Inflammations' or Inflammation, Lung or 'pulmonary embolism*' or 'Embolism, Pulmonary' or 'Pulmonary Thromboembolisms' or 'Thromboembolism, Pulmonary' or 'Thromboembolisms, Pulmonary' or 'Pulmonary Edema*' or 'Wet Lung or Lung, Wet' or 'Pulmonary Edemas' or 'Edema, Pulmonary' or 'Respiratory Insufficiency*' or Acute Hypercapnic Respiratory Failure or Hypercapnic Acute Respiratory Failure or Respiratory Failure or Hypoxemic Respiratory Failure or Failure, Hypercapnic Respiratory or Respiratory Depression or 'Bronchitis*' or Bronchiolitis, Viral or Bronchiolitis or 'Asthma*' or Asthmas or Bronchial Asthma or Asthma, Bronchial) and (adult*)) 4005 |

Note：Deadline is January 8, 2025

**Supplementary Table 2: Baseline information**

| **Study** | **Baseline**  **HbA1c (%)** | **Baseline**  **BMI (kg/m^2^)** | **Baseline**  **eGFR(mL/min/1.73m^2^)** | **Duration of diabetes (years)** |
| --- | --- | --- | --- | --- |
| White (2013) | 8 | 28.7 | 71.2 | 7.0 |
| Scirica (2013) | 8 | 31.2 | 72.6 | 10.3 |
| Green (2015) | 7.2 | 30.2 | 74.9 | 12.0 |
| Gantz ( 2017) | 8 | 31.3 | 86.2 | 12.0 |
| Rosenstock (2019)a | 7.9 | 31.3 | 54.7 | 15.0 |
| Rosenstock (2019)b | 7.2 | 30.1 | 76.7 | 6.0 |
| Pfeffer 2015 | 7.6 | 30.1 | 76 | 9.3 |
| Marso (2016)a | 8.7 | 32.5 | NR | 12.9 |
| Marso (2016)b | 8.7 | 32.8 | NR | 13.9 |
| Holman (2017) | 8 | 31.8 | 76.3 | 12.0 |
| Hernandez (2018) | 8.7 | 32.3 | 79 | 14.0 |
| Gerstein (2019) | 7.3 | 32.3 | 75 | 11.0 |
| Husain(2019) | 8.2 | 32.3 | 74 | 15.0 |
| Gerstern(2021) | 8.91 | 32.7 | 72.4 | 11.0 |
| Wanner (2016) | 8.1 | 30.6 | 74.1 | NR |
| Wiviott (2018) | 8.3 | 32 | 76.5 | 11.0 |
| Mahaffey (2018)a | 8.2 | 31.9 | 76.7 | 13.5 |
| Mahaffey (2018)b | 8.2 | 31.9 | 76.7 | 13.5 |
| Perkovic (2019) | 8.3 | 31.3 | 56.2 | 16.0 |
| Cannon (2020) | 8.2 | 32.0 | 75.9 | 13.0 |
| Bhatt( 2021)a | 8.3 | 31.9 | NR | NR |
| McMurray (2019) | NR | 28.2 | 65.4 | NR |
| Heerspink (2020) | NR | 29.5 | 43.1 | NR |
| Packer 2020 | NR | 28.0 | 61.8 | NR |
| Anker 2021 | NR | 29.77 | 60.6 | NR |
| Peikert 2022 | NR | NR | 62 | NR |
| Herrington 2023 | NR | 29.7 | NR | NR |

**Supplementary Figure 1: Number of trials evaluating DPP-4 inhibitors, GLP-1RAs,and SGLT2 inhibitors for risk of eight respiratory diseases.**

**Supplementary Figure 2: Bias risk assessment for trials.**


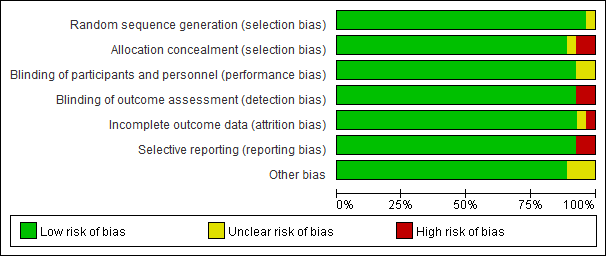


**Supplementary Figure 3: COPD**

The impact of new hypoglycemic drugs and drug subtypes on the risk of COPD. (1) Forest plot of pairwise meta-analysis on the impact of novel antidiabetic medications on COPD risk. (2) Forest plot of network meta-analysis on the impact of novel antidiabetic medications on COPD risk. (3) The funnel plot is used to publish bias. (4) SUCRA plot for all treatments. (5) Subgroup pairwise meta-analysis (T2DM vs mixed population). (6) Subgroup pairwise meta-analysis (drug types). (7) Subgroup analysis: Network meta-analysis of forest maps. (8) Subgroup analysis: funnel plot. (9) Subgroup analysis: Using the SUCRA plot to evaluate the safest medication for intervening in COPD.

(1) (2) (3)

(4) (5) (6)

(7) (8) (9)

**Supplementary Figure 4: Pneumonia**

The impact of new hypoglycemic drugs and drug subtypes on the risk of pneumonia. (1) The forest plot for pairwise meta-analysis of the impact of novel antidiabetic medications on pneumonia risk is being presented. (2) The forest plot for network meta-analysis of the impact of novel antidiabetic medications on pneumonia risk. (3) The funnel plot is used to publish bias. (4) Using the SUCRA plot to assess the safest intervention among novel antidiabetic medications for pneumonia. (5) Forest map of association between diabetes and pneumonia risk. (6) Subgroup analysis: Forest maps for pairwise meta-analysis. (7) Subgroup analysis: Network meta-analysis of forest maps. (8) Subgroup analysis: funnel plot. (9) Subgroup analysis: Using the SUCRA plot to evaluate the safest medication for intervening in pneumonia.

(1) (2) (3)

(4) (5) (6)


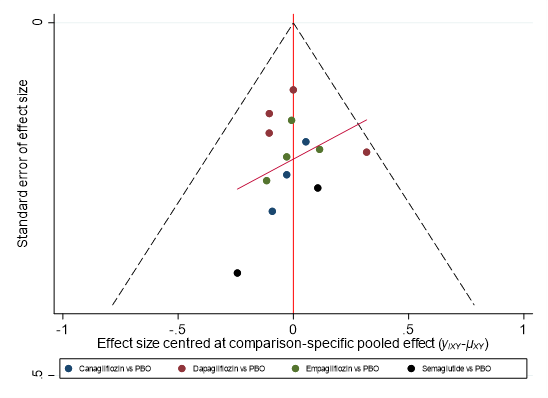


(7) (8) (9)

**Supplementary Figure 5: Pulmonary edema**

The impact of new hypoglycemic drugs and drug subtypes on the risk of pulmonary edema. (1) Forest plot of pairwise meta-analysis on the impact of novel antidiabetic medications on Pulmonary edema risk. (2) Forest map of a network meta-analysis of the effects of novel hypoglycemic drugs on the risk of pulmonary edema. (3) The funnel plot is used to publish bias. (4) Using SUCRA plots to evaluate the safest drug intervention for pulmonary edema among new hypoglycemic medications. (5) Forest map of risk association between diabetes and pulmonary edema. (6) Subgroup analysis: Forest maps for pairwise meta-analysis. (7) Subgroup analysis: Network meta-analysis of forest maps. (8) Subgroup analysis: funnel plot. (9) Subgroup analysis: Using the SUCRA plot to evaluate the safest medication for intervening in pulmonary edema.

(1) (2) (3)

(4) (5) (6)

(7) (8) (9)

**Supplementary Figure 6:** **Pulmonary embolism**

The impact of new hypoglycemic drugs and drug subtypes on the risk of pulmonary embolism. (1) Forest plot of paired meta-analysis on the impact of novel antidiabetic medications on the risk of pulmonary embolism. (2) Forest map of a network meta-analysis of the effects of novel hypoglycemic drugs on the risk of pulmonary embolism. (3) The funnel plot is used to publish bias. (4) Using SUCRA plots to evaluate the safest drug intervention for embolism among new hypoglycemic medications. (5) Forest map of association between diabetes mellitus and pulmonary embolism risk. (6) Subgroup analysis: Forest maps for pairwise meta-analysis. (7) Subgroup analysis: Network meta-analysis of forest maps. (8) Subgroup analysis: funnel plot. (9) Subgroup analysis: Using the SUCRA plot to evaluate the safest medication for intervening in pulmonary embolism.

(1) (2) (3)

(4) (5) (6)

(7) (8) (9)

**Supplementary Figure 7:** **Respiratory failure**

The impact of new hypoglycemic drugs and drug subtypes on the risk of respiratory failure. (1) Forest plot of paired meta-analysis on the impact of novel antidiabetic medications on the risk of respiratory failure. (2) Forest plot of a network meta-analysis on the impact of new hypoglycemic drugs on the risk of respiratory failure. (3) The funnel plot is used to publish bias. (4) Using SUCRA plots to evaluate the safest drug intervention for respiratory failure among new hypoglycemic medications. (5) Forest map of association between diabetes and respiratory failure risk. (6) Subgroup analysis: Forest maps for pairwise meta-analysis. (7) Subgroup analysis: Network meta-analysis of forest maps. (8) Subgroup analysis: funnel plot. (9) Subgroup analysis: Using the SUCRA plot to evaluate the safest medication for intervening in respiratory failure.

(1) (2) (3)

(4) (5) (6)

(7) (8) (9)

**Supplementary Figure 8: Bronchitis**

The impact of new hypoglycemic drugs and drug subtypes on the risk of pneumonia. (1) Forest plot of paired meta-analysis on the impact of novel antidiabetic medications on the risk of bronchitis. (2) Forest plot of a network meta-analysis on the impact of new hypoglycemic drugs on the risk of bronchitis. (3) The funnel plot is used to publish bias. (4) Using SUCRA plots to evaluate the safest drug intervention for bronchitis among new hypoglycemic medications. (5) Forest map of association between diabetes and bronchitis risk. (6) Subgroup analysis: Forest maps for pairwise meta-analysis. (7) Subgroup analysis: Network meta-analysis of forest maps. (8) Subgroup analysis: funnel plot. (9) Subgroup analysis: Using the SUCRA plot to evaluate the safest medication for intervening in bronchitis.

(1) (2) (3)

(4) (5) (6)

(7) (8) (9)

**Supplemental Figure 9:** **Asthma**

The impact of new hypoglycemic drugs and drug subtypes on the risk of asthma. (1) Forest plot of paired meta-analysis on the impact of novel antidiabetic medications on the risk of bronchitis. (2) Forest plot of a network meta-analysis on the impact of new hypoglycemic drugs on the risk of asthma. (3) The funnel plot is used to publish bias. (4) Using SUCRA plots to evaluate the safest drug intervention for asthma among new hypoglycemic medications. (5) Forest map of association between diabetes and asthma risk. (6) Subgroup analysis: Forest maps for pairwise meta-analysis. (7) Subgroup analysis: Network meta-analysis of forest maps. (8) Subgroup analysis: funnel plot. (9) Subgroup analysis: Using the SUCRA plot to evaluate the safest medication for intervening in asthma.

(1) (2) (3)

(4) (5) (6)

(7) (8) (9)

**Supplementary Figure 10**: The paired meta-analysis forest plot for newly added obese patients. (1) COPD; (2) Pneumonia; (3) Respiratory failure; (4) Bronchitis; (5) Asthma; (6) Pulmonary embolism; (7) Pulmonary edema.


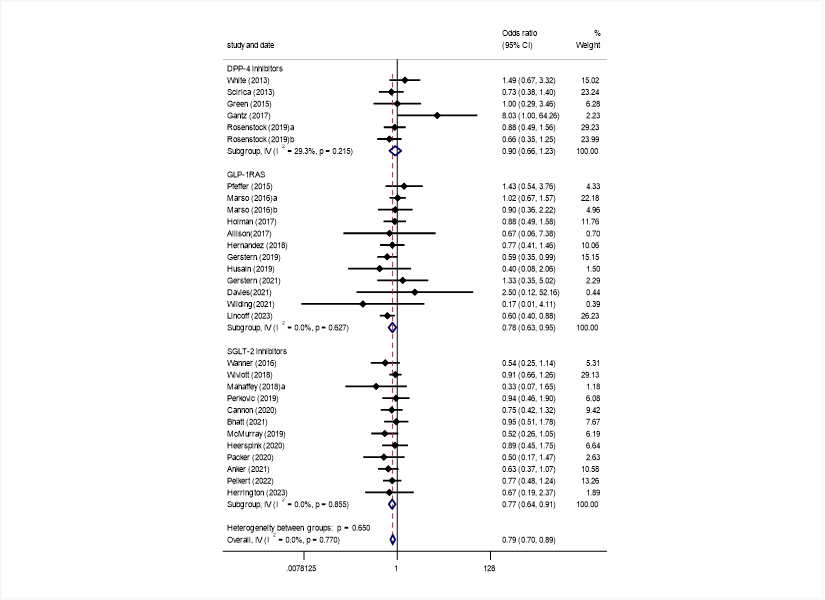


(1) (2) (3)


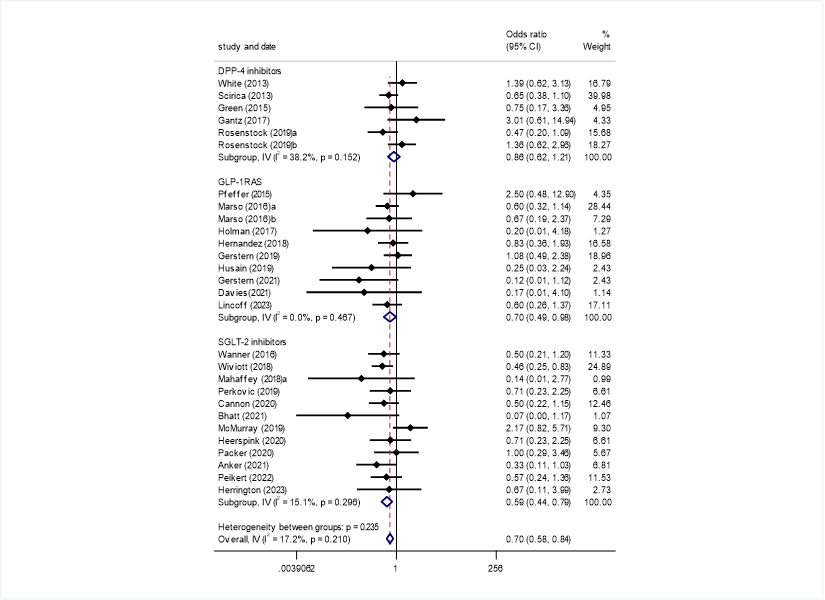

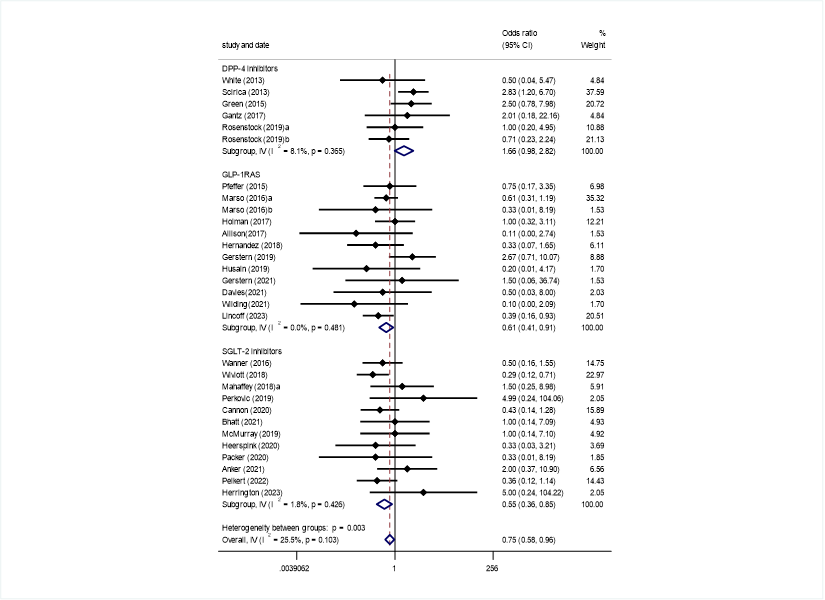

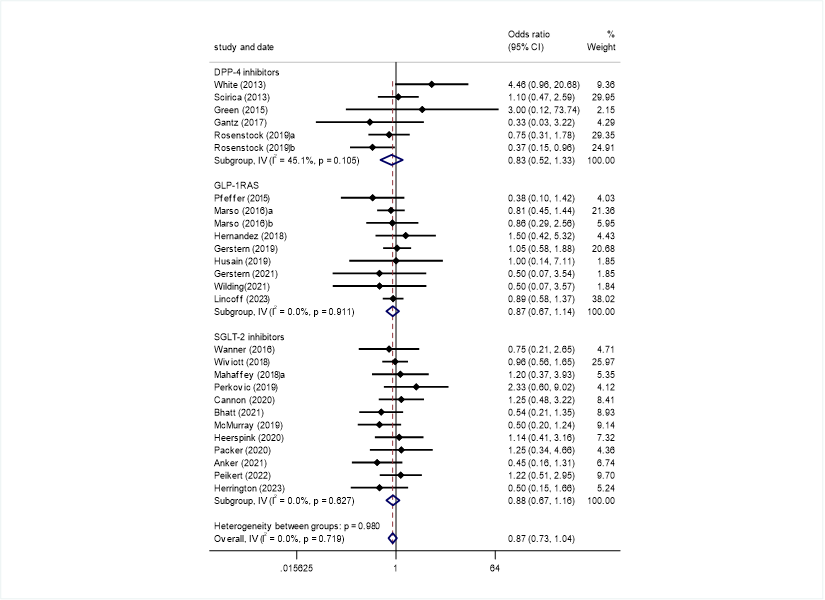


(4) (5) (6)


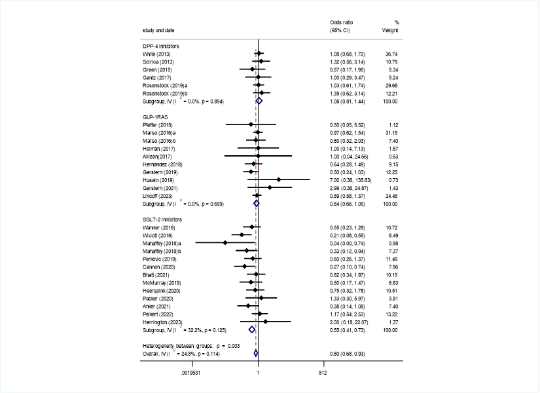


(7)

**Supplementary Figure 11**: A comparison of the effects of GLP-1RAs on respiratory diseases in two types of patients. GLP-1RAs represent the group of patients with type 2 diabetes, while GLP-1RAs' represent the group of obese patients. (1) Pneumonia; (2) Bronchitis; (3) Respiratory failure; (4) Asthma; (5) Pulmonary embolism.


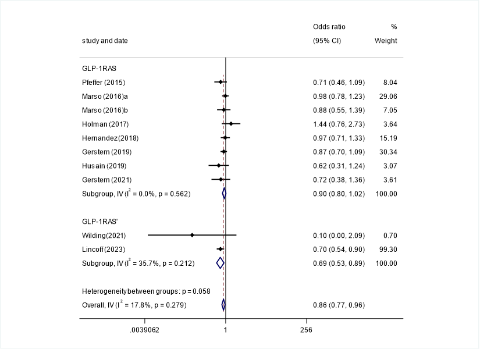

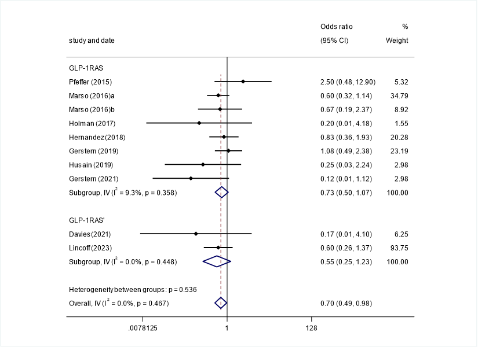

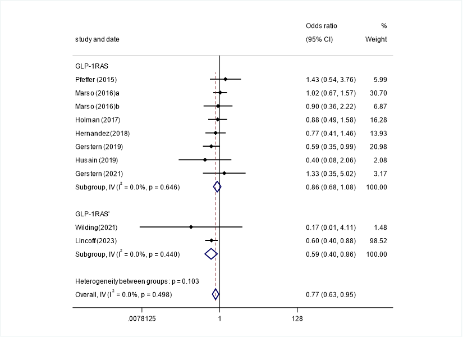


(1) (2) (3)


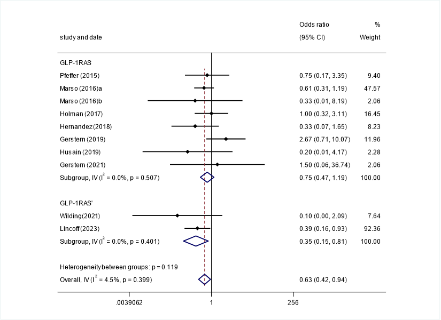

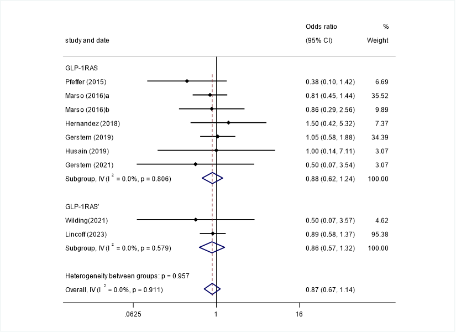


(4) (5)

**Supplementary Table 3**: A search strategy for newly added obese patients in the GLP-1RAs group. Search policy

| Pubmed Search terms |
| --- |
| ((‘Glucagon-Like Peptide-1 Receptor’[Mesh] OR ‘Glucagon-like peptide 1 receptor agonist*’ OR ‘GLP-1 receptor agonist*’ OR ‘GLP-1 agonist*‘ OR ‘GLP-1RA’ OR exenatide OR liraglutide OR lixisenatide OR albiglutide OR dulaglutide OR semaglutide) AND (‘Diabetes Mellitus, Type 2’[Mesh] OR ‘Type 2 diabetes’ OR T2DM OR T2D OR 'Obesity'[Mesh] OR Overweight ) AND ("Randomized Controlled Trial"[Publication Type] OR random OR RCT OR placebo OR trial) AND (Cardiovascular OR renal OR CVOT OR 'CVOT' OR HF OR CKD OR ‘Pulmonary Disease, Chronic Obstructive’[MeSH] OR ‘Chronic Obstructive Airway Disease’ OR ‘Chronic Obstructive Lung Disease’ OR ‘COAD’ OR ‘Chronic Obstructive Pulmonary Diseases’ OR ‘COPD’ OR ‘Chronic Obstructive Pulmonary Disease’ OR ‘Airflow Obstructions, Chronic’ OR ‘Airflow Obstruction, Chronic’ OR ‘Chronic Airflow Obstruction’ OR ‘Chronic Airflow Obstructions’ OR ‘Chronic Obstructive Pulmonary Disease’ OR ‘Pneumonias’[MeSH] OR ‘Lobar Pneumonia’ OR ‘Lobar Pneumonias’ OR ‘Pneumonias, Lobar’ OR ‘Pneumonia, Lobar’ OR ‘Experimental Lung Inflammation’ OR ‘Inflammation, Experimental Lung’ OR ‘Lung Inflammation’ OR ‘Pulmonary Inflammations’ OR Inflammation, Lung OR ‘pulmonary embolism’[MeSH] OR ‘Embolism, Pulmonary’ OR ‘Pulmonary Thromboembolisms’ OR ‘Thromboembolism, Pulmonary’ OR ‘Thromboembolisms, Pulmonary’ OR ‘Pulmonary Edema’[MeSH] OR ‘Wet Lung OR Lung, Wet’ OR ‘Pulmonary Edemas’ OR ‘Edema, Pulmonary’ OR 'Respiratory Insufficiency'[MeSH] OR Acute Hypercapnic Respiratory Failure OR Hypercapnic Acute Respiratory Failure OR Respiratory Failure OR Hypoxemic Respiratory Failure OR Failure, Hypercapnic Respiratory OR Respiratory Depression OR 'Bronchitis'[MeSH] OR Bronchiolitis, Viral OR Bronchiolitis OR 'Asthma'[MeSH] OR Asthmas OR Bronchial Asthma OR Asthma, Bronchial) AND (alladult[Filter])) 562 |

Note：Deadline is January 8, 2025

**Supplementary Table 4**: The baseline table for patients with obesity or overweight.

| Study | NCT ID | Name | Population | Comparison | Trial duration | Total | Average age(years) | BMI  (kg/m2) | Mean  HbA1c(%) |
| --- | --- | --- | --- | --- | --- | --- | --- | --- | --- |
| Davies(2021) | NCT03552757 | STEP 2 | T2DM and overweight or obesity | Semaglutide  VS  Placebo | 68  week | 1210 | 55.0 | 35.7 | 8·1 |
| Wilding(2021) | NCT03548935 | STEP 1 | Overweight or Obesity | Semaglutide  VS  Placebo | 68  week | 1961 | 46.0 | 37.9 | 5.7 |
| Lincoff(2023) | NCT03574597 | SELECT | Overweight or Obesity | Semaglutide  VS  Placebo | 68  week | 17,604 | 46.0 | 37.8 | 5.7 |

**Supplementary Table 5**: Distribution of participants with and without type 2 diabetes in SGLT2 inhibitor trials enrolling mixed populations.

| Study | NCT ID | Name | T2DM  Population | Without  T2DM  Population | Total |
| --- | --- | --- | --- | --- | --- |
| McMurray (2019) | NCT03036124 | DAPA-HF | 1983 | 2761 | 4744 |
| Heerspink (2020) | NCT03036150 | DAPA-CKD | 2906 | 1398 | 4304 |
| Packer (2020) | NCT03057977 | EMPEROR-Reduced 2020 | 1856 | 1874 | 3730 |
| Anker (2021) | NCT03057951 | EMPEROR-Preserved | 2938 | 3050 | 5988 |
| Peikert (2022) | NCT03619213 | EMPEROR-Preserved | 2806 | 3457 | 6263 |
| Herrington (2023) | NCT03619213 | EMPA-KIDNEY | 2936 | 3673 | 6609 |
